# The influence of maternal gut and vaginal microbiota on gastrointestinal colonization of neonates born vaginally and per caesarean section

**DOI:** 10.1186/s12884-025-07358-w

**Published:** 2025-03-08

**Authors:** Emma Ronde, Maaike Alkema, Thomas Dierikx, Sam Schoenmakers, Clara Belzer, Tim de Meij

**Affiliations:** 1 Division of Obstetrics and Prenatal Diagnosis, Erasmus University Medical Centre, Dr. Molewaterplein 40, Rotterdam, 3015 GD The Netherlands; 2 Laboratory of Microbiology, Wageningen University, Dreijenplein 10, Wageningen, 6703 HB The Netherlands; 3 Department of Medical Microbiology, Infectious Diseases & Infection Prevention, Maastricht University Medical Center, Maastricht, The Netherlands; 4 Department of Pediatric Gastroenterology, Emma Children’s Hospital, Amsterdam UMC, Meibergdreef 9, Amsterdam, 1105 AZ The Netherlands

**Keywords:** Microbiota, Colonization, Seeding, Neonatal microbiota, Microbial transmission, Caesarean section

## Abstract

**Background:**

Early life microbial colonization of the neonatal gastrointestinal tract is crucial for imprinting of the immune system. Vertical transmission of maternal microbes is considered the key source of initial neonatal microbial colonization. We aimed to evaluate the role of the maternal vaginal and rectal microbiota in early neonatal gastrointestinal colonization in vaginally- and caesarean section-born neonates.

**Methods:**

Maternal vaginal and rectal swabs were collected shortly before delivery. Neonatal fecal samples were collected at day 0, 7 and 28 postnatally in both vaginally-born (*n* = 23) and caesarean-section born (*n* = 40) neonates (total *n* = 63). All samples were analyzed by 16 S rRNA sequencing. The relative abundances of amplicon sequence variants (ASVs) shared between maternal swabs and fecal neonatal samples were compared in vaginally-versus caesarean section-born neonates.

**Results:**

The median relative abundance of ASVs shared in the maternal rectal and vaginal swabs with all neonatal samples was low (below 10% for rectal or vaginal swabs with any of the three time-points). When focusing on vaginally- versus caesarean section-born neonates, there were no differences in the relative abundance of shared ASVs with the maternal vaginal swabs, and only on day 7 in the rectal swabs (*p* = 0.002). However, in both delivery routes, the relative abundance of ASV shared with the maternal rectal swab was higher (median 19% in vaginally-born neonates and 2% in caesarean section-born neonates) compared to the relative abundance of the ASVs shared with the maternal vaginal swab (0% for both vaginally- and caesarean section-born neonates) on day 28.

**Conclusions:**

We observed that only a limited amount of ASVs were transferred from maternal rectal and vaginal compartments to the neonatal gastrointestinal tract. ASVs from the maternal gastrointestinal tract contributed to neonatal gut colonization to a greater extent than ASVs from the maternal genital tract at one month of age. Our findings contribute to an increased understanding of factors influencing neonatal gastrointestinal colonization in both caesarean section and vaginal birth, of importance as characteristics of early colonization have been associated with health outcomes later in life.

**Trial registration:**

The original trial is registered with the Dutch Clinical Trial Registry (Trial registration number: NTR6000, https://www.trialregisternl/trial/5845) and the study protocol was published online.

**Supplementary Information:**

The online version contains supplementary material available at 10.1186/s12884-025-07358-w.

## Background

The human gut microbiota harbors around 10^13^–10^14^ microorganisms including bacteria, viruses and fungi [1]. Neonatal colonization is considered to initiate with vertical transmission of microbes from mother to offspring during delivery, followed by horizontal transmission (through non-hereditary means) during the first days and weeks of life. The characteristics of initial colonization can differ, and affect proper development of the gastrointestinal tract and imprinting of the immune system [2]. Disruption of the physiological colonization process, for example by caesarean section and/or antibiotics, has previously been associated with an increased risk of a variety of diseases beyond neonatal age during the entire life course, including obesity, allergies, asthma and inflammatory bowel disease [2, 3]. Postnatal colonization in both vaginally-born and caesarean-born neonates depends on a wide spectrum of environmental factors, such as the method of feeding (breast feeding or formula), parental and peer exposure, and exposure to medication, particularly antibiotics [4–6]. 

Vaginally-born (VB) neonates are initially exposed to mainly maternal vaginal and perineal microbes, whereas neonates born per caesarean section are not subject to this physiological process and mainly exposed to maternal skin microbes during birth and the postpartum period of breastfeeding [7]. Previous studies have shown that accordingly, vaginally-born neonates show an intestinal predominance of *Lactobacillus* and *Bifidobacterium*, likely transmitted from their mothers [7]. In contrast, neonates born by caesarean section (CS) are characterized by an aberrant intestinal microbial colonization, even beyond the first year of age, compared to neonates born vaginally, characterized by a decreased abundance of *Bacteroides* species [8–10]. Decreased microbial diversity has been observed up to even two years of age in offspring born via CS versus those born vaginally [9, 10]. 

The exact mechanism of vertical microbial seeding from perineal, rectal and vaginal sources to the neonatal gastrointestinal tract, is yet poorly understood. Recent studies have shown that, despite a high relative abundance of, for example *Lactobacillus* in maternal vaginal microbiota, the transmission of other microbes from maternal rectal sources may play a more significant role in vertical transmission [5, 11]. Detailed knowledge on these mechanisms could improve insight into the development of the neonatal gut microbiota colonization and may result in the development of novel microbiota-based strategies to prevent or resolve early dysbiosis, which is associated with negative health outcomes even beyond infancy.

The aim of this study was to assess how maternal vaginal and rectal microbiota contribute to colonization of the neonatal gastrointestinal tract, both in vaginally-and CS-born neonates.

## Methods

### The aim, design and setting of the study

This study is a retrospective cohort study. For this study, 63 mother-neonate pairs included in a previous randomized controlled trial were eligible to participate in this study [12]. The aim of the previous study was to assess the influence of timing of prophylactic antibiotics in CS (administered maternally prior to skin incision versus after clamping of the umbilical cord postpartum) on neonatal gut microbial colonization, up to three years of age. In total, 40 neonates born per CS (20 mothers randomized to receiving prophylactic cefuroxime 1500 mg intravenously prior to skin incision and 20 after clamping of the umbilical cord) were included and 23 vaginally-born (VB) neonates as control group. The vaginally born neonates did not receive any antibiotics during 28 days postpartum or during birth. Nor did their mothers receive any antibiotics during pregnancy, delivery or 28 postpartum. All 63 neonates were born at term and did not receive antibiotic or immunosuppressive medication during the first 28 days postpartum. Beside prophylactic antibiotics during CS, none of the included mothers received any antibiotics during pregnancy, delivery or the first month postpartum.

Pregnant women with a normal-weight fetus, delivering at gestational age above 37 weeks scheduled for a primary CS were eligible to participate in this study.

Exclusion criteria are displayed in the published study protocol and can also be found in Table 1 [12]. For the purpose of the current study, we additionally collected vaginal and rectal swabs of the mothers, within 24 h before delivery. The study protocol was approved by the research ethics review committee Faculty of Science Vrije Universiteit Medical Center (2014.468). Written informed consent was given by both parent of all included participants. The original trial is registered with the Dutch Clinical Trial Registry in accordance with the Declaration of Helsinki (Trial registration number: NTR6000, https://www.trialregisternl/trial/5845) and the study protocol was published online [12]. 

**Table 1:** Exclusion criteria

Maternal	Neonatal		
Delivery < 37 weeks gestation Aged ≤ 17 years Hypertensive pregnancy disorder Multiple pregnancy Body mass index (BMI) ≥ 25 Antibiotic use during pregnancy Antibiotic use during first month postpartum Immunosuppressive usage within 3 months prior to delivery Inflammatory bowel disease Celiac disease Rupture of membranes before caesarean section Prolonged rupture of membranes for > 18 h Diabetes mellitus type I/II Gestational diabetes requiring insulin History of major gastro-intestinal surgery Alcohol or tobacco use in second and third trimester Drug use during pregnancy		Small or large for gestational age Congenital gastro-intestinal anomalies Gastro-intestinal surgery during the first month of life Antibiotic medication during the first month of life Immunosuppressive medication use during the first month of life	

### Sample collection

A maternal vaginal and rectal swab (Copan flocked swabs with eNat buffer) was obtained upon admission when the woman presented in labor at the delivery ward and directly stored at -20 °C. In caesarean section delivering women, a maternal vaginal and rectal swab was obtained upon placing the urine catheter during the caesarean section and directly stored at -20 °C until further handling. The neonate’s first stool (meconium) was collected on day 0 postpartum in a sterile container (Stuhlgefäß 10mL, Frickenhausen, Germany) by the nurse or midwife from the diaper and stored at -20 °C. Neonatal stool samples at day 7 and 28 after birth were collected by parents at home in similar containers. Parents obtained instructions to use the sampling tool provided within it to scoop meconium from the diaper and place it immediately into the container to prevent any contamination. They were then instructed to immediately close the lid and place the sample in the freezer.

**Fig. 1:**
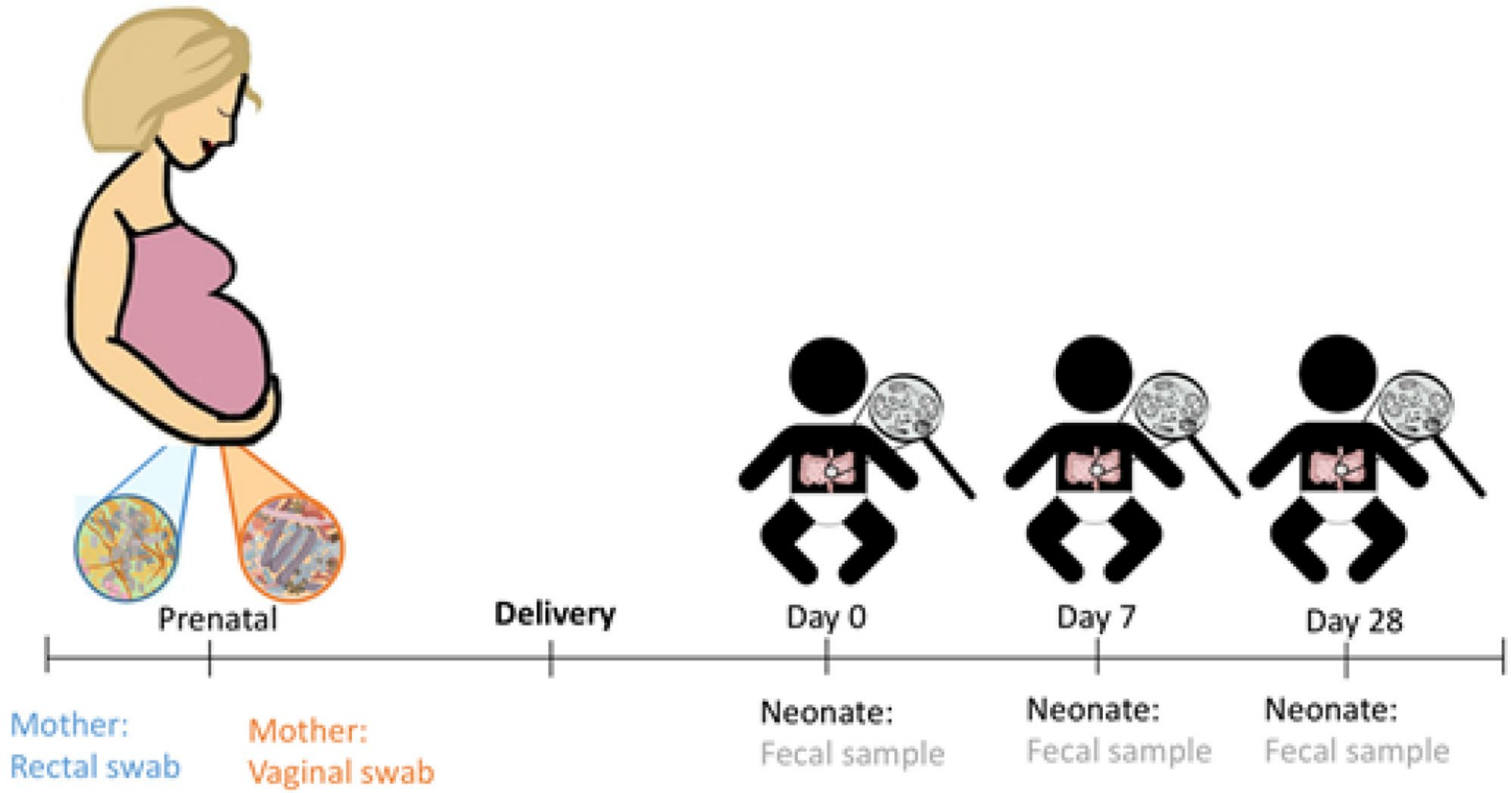
Timeline and type of maternal and neonatal sample

These samples were, transported in frozen condition to the hospital on the day of the regular postpartum check-up 6 weeks postpartum and stored at -20 °C until analysis. An outline of the study including the timing of sampling is demonstrated in Fig. 1.

### Sample handling

The neonatal fecal samples from day 0, day 7 and day 28 were previously sequenced for another trial [9]. For the current study, we used that previously sequenced data. The same protocols were used for DNA extraction and sequencing of the maternal vaginal and rectal swabs. Briefly, DNA was extracted using the NucliSENS easyMag (bioMérieux, Marcy l’Etoile, France). The V3–V4 hypervariable regions of the bacterial 16 S rRNA gene were amplified (341 F-785R). These sequences were used for the amplicon sequence variant analyses. Universal primers S-D-Bact-0341-b-S-17 and S-D-Bact-0785-a-A-21 were used for amplification. Sequencing was performed on an Illumina MiSeq instrument (Illumina, San Diego, California, USA) using the 2 × 300 bp paired-end sequencing protocol by LifeSequencing S.L. (Valencia, Spain). A detailed description of the DNA extraction and sequencing can be found in the previously published paper [9]. Demultiplexed reads were filtered using the NG-Tax pipeline with default parameters [13–15]. Taxonomic assignment was done in NG-Tax using the default parameters and the SILVA database [16]. The number of species found in each sample reached a plateau in the rarefaction curve (supplementary Fig. 1), indicating that samples were sufficiently sequenced. We decided not to rarefy the samples to even depth, since we aimed to assess the presence of shared amplicon sequence variants (ASVs) in mother- and neonate pairs, and rarefying the samples to even depth might have resulted in loss of some ASVs.

### Microbiota analysis

The microbiota analysis consisted of two parts:

1.) General, descriptive analysis of microbiota

The number of ASVs and relative abundance at family level of maternal vaginal and rectal swabs were assessed. The same was done for neonatal samples (for vaginally- and caesarean-born neonates) at the three predefined time points (d0, d7 and d28). In maternal vaginal swabs and rectal swabs a median of 26 (41551 reads) and 83 (43764 reads) ASVs were identified respectively. In the neonatal samples a total median number of 39 (reads 43522), 24 (40710 reads) and 23 ASVs (50273 reads) were found at day 0, 7 and 28 respectively.

2.) Analysis of maternal ASV transmission

A. *Assessment of amount of maternal transfer from vaginal microbiota versus rectal microbiota*: In order to compare the role of the maternal vaginal microbiota with the role of the rectal microbiota, the relative abundances of ASVs in the neonatal microbiota shared with maternal vaginal swabs was compared to the relative abundance shared with maternal rectal swabs. This was done for vaginally-born and caesarean-born neonates separately.
B. *Impact of route of delivery on transmission of ASVs from mother to neonate*: Shared ASVs were defined as ASV present in both the maternal vaginal and/or rectal swab and in the neonatal sample in a mother-neonate pair. First, ASVs shared between the maternal vaginal swabs and neonatal fecal samples were identified. The percentage of shared ASVs out of total ASVs per sample were calculated for vaginally-born and caesarean-born neonates separately. The relative abundances of these shared ASVs were calculated. In order to detect differences between both delivery routes in the role of the maternal vaginal microbiota on neonatal gut colonization, we compared the relative abundance of ASVs shared with the maternal vaginal microbiota in vaginally-born neonates to the relative abundance in CS-born neonates. Shapiro test was done to confirm that data was not normally distributed. Mann-Whitney-U test was done to compare the relative abundance of shared ASVs between groups. The same was done for ASVs shared between the maternal fecal swabs and the neonatal fecal samples. A p-value of < 0.05 was considered significant.

## Results

### Study population

A total of 63 mother-neonate pairs included in the original randomized controlled trial participated in this study [12]. Baseline characteristics of included mothers are shown in Table 2. All neonates were born at term, but vaginally-born neonates had a significantly higher gestational age. Also, caesarean section born neonates had significantly older mothers.

**Table 2:** Baseline characteristics

Clinical Values	Caesarean-section born (*n* = 40)	Vaginally-born (*n* = 23)	*P*-value
Gestational age, median [IQR], weeks + days	39 + 0 [38 + 6–39 + 1]	39 + 6 [38 + 4–40 + 6]	0.02
Birthweight, mean (sd), grams	3840 (493)	3385 (484)	0.46
Female sex, n (%)	19 (48)	14 (61)	0.31
Maternal age at birth, median [IQR], years	36.1 [32.6–39.1]	32.3 [30.8–35.9]	0.01
BMI, median [IQR], kg/m²	23.1 [21.0–24.7]	21.9 [20.8–23.3]	0.20
Gravida, median [IQR]	3 [2–4]	2 [1–3]	0.37
Para, median [IQR]	1 [1–1]	1 [0–1]	0.34
Maternal diet at birth, n (%)			0.41
Vegetarian	37 (93)	20 (87)	
Non-vegetarian	2 (5)	3 (13)	
Missing	1 (3)	0 (0)	
Maternal probiotic use, n (%)	0 (0)	1 (4)	0.18
Neonatal probiotic use, n (%)	0 (0)	0 (0)	1.00
1-minute APGAR score, median [IQR]	9 [9–9]	9 [8–9]	0.16
5-minute APGAR-score, median [IQR]	10 [10–10]	10 [9–10]	0.17
Meconium stained amniotic fluid, n (%)	1 (3)	3 (13)	0.10
Feeding type, n (%)			0.49
Breastfed	20 (50)	15 (65)	
Formula fed	9 (23)	4 (17)	
Combination	11 (28)	4 (17)	

IQR: interquartile range; sd (standard deviation)

#### Assessment of amount of maternal transfer from vaginal microbiota versus rectal microbiota to fecal samples

**Fig. 2:**
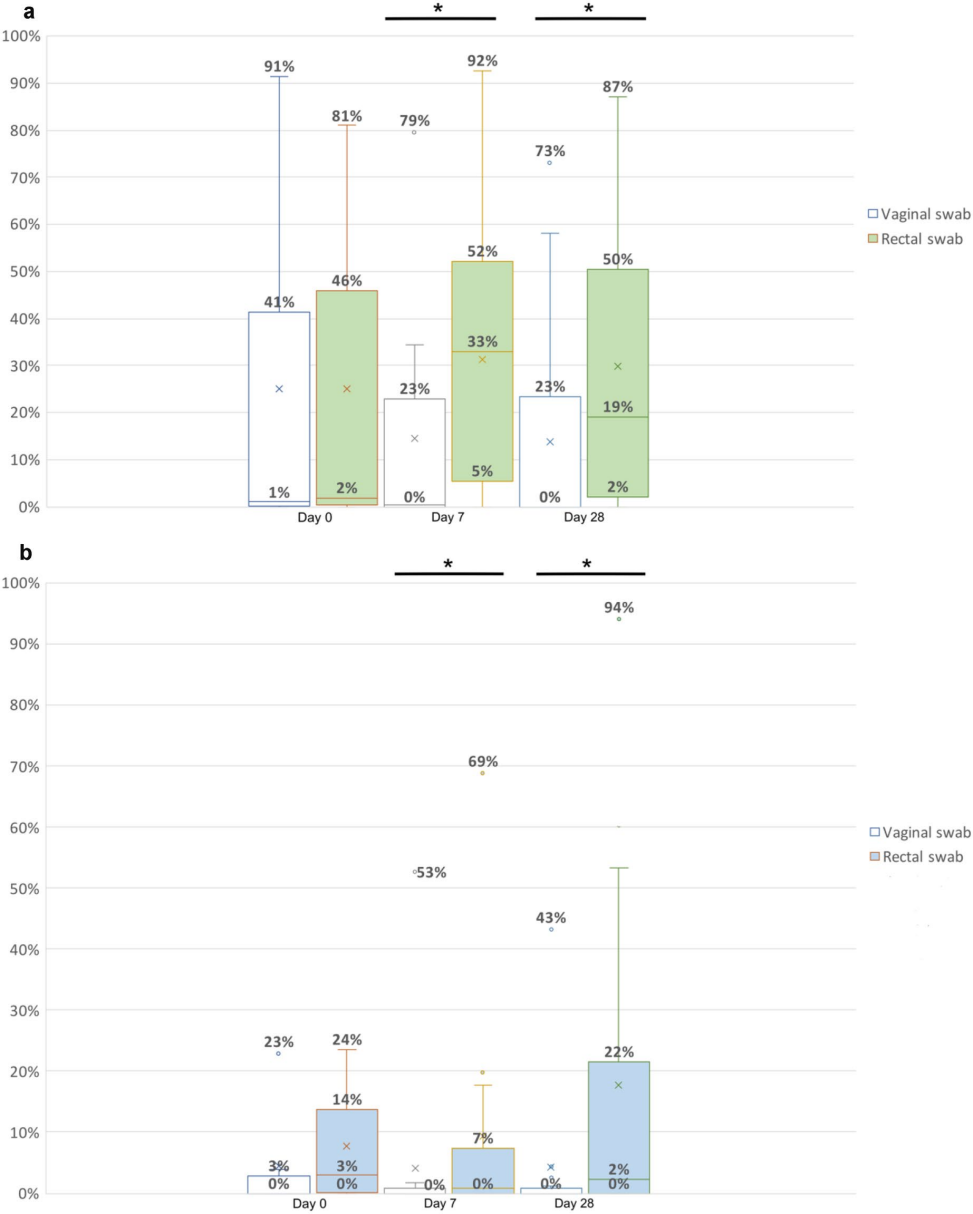
**a** Relative abundance of shared ASVs between maternal vaginal versus rectal samples in VB-neonates. Day 0: vaginal swab versus rectal swab *p* = 0.902, Day 7: vaginal swab versus rectal swab *p* = 0.024, Day 28: vaginal swab versus rectal swab *p* = 0.022. The asterisk represents significance. **b** Relative abundance of shared ASVs between maternal vaginal versus rectal samples in CS neonates. Day 0: vaginal swab versus rectal swab *p* = 0.394, Day 7: vaginal swab versus rectal swab *p* = 0.034, Day 28: vaginal swab versus rectal swab: 0.014. The asterisk represents significance

In vaginally-born neonates a median of 1% (IQ range 0–41%, range 0–91%) of the ASVs was present in the maternal vaginal swab and found in the neonatal fecal sample on day 0. On day 7 a median of 0% (IQ range 0–23%, range 0–79%) of the ASVs was present in the maternal vaginal swab and found in the neonatal fecal sample and a median of 0% (IQ range 0–23%, range 0–73%) of the ASVs was present in the neonatal fecal sample on day 28 and also found in the maternal vaginal swab (white bars in Fig. 2A).

In vaginally-born neonates a median of 2% (IQ range 0–46%, range 0–81%) of the ASVs was present in the maternal rectal swab and found in the neonatal fecal sample on day 0. On day 7 a median of 33% (IQ range 5–52%, range 0–92%) of the ASVs was present in the maternal rectal swab and found in the neonatal fecal sample and a median of 19% (IQ range 2–50%, range 0–87%) of the ASVs was present in the neonatal fecal sample on day 28 and also found in the maternal rectal swab (green bars in Fig. 2A). When comparing the relative abundance of shared ASVs between the vaginal swab with the rectal swab in VB-neonates, there were no differences on day 0 (*p* = 0.902), however there was a significant difference both on day 7 (*p* = 0.024) and day 28 (*p* = 0.022).

In caesarean section born neonates a median of 0% (IQ range 0–3%, range 0–23%) of the ASVs was present in the maternal vaginal swab and found in the neonatal fecal sample on day 0. On day 7 a median of 0% (IQ range 0–0%, range 0–53%) of the ASVs was present in the maternal vaginal swab and found in the neonatal fecal sample and a median of 0% (IQ range 0–0%, range 0–43%) of the ASVs was present in the neonatal fecal sample on day 28 and also found in the maternal vaginal swab (white bars in Fig. 2B).

In caesarean section born neonates a median of 3% (IQ range 0–14%, range 0–24%) of the ASVs was present in the maternal rectal swab and found in the neonatal fecal sample on day 0. On day 7 a median of 0% (IQ range 0–7%, range 0–69%) of the ASVs was present in the maternal rectal swab and found in the neonatal fecal sample and a median of 2% (IQ range 0–22%, range 0–94%) of the ASVs was present in the neonatal fecal sample on day 28 and also found in the maternal rectal swab (blue bars in Fig. 2B). When comparing the relative abundance of shared ASVs between maternal vaginal versus rectal samples in CS neonates, we did not observe a significant difference on day 0 (*p* = 0.394), however there was a significant difference both on day 7 (*p* = 0.034) and day 28 (*p* = 0.014.)

We observed that ASVs shared between the neonate and maternal rectal swab typically came from the genus *Bacteroides* and *Bifidobacterium.* The only detected ASVs shared with the maternal vaginal swabs included *Streptococci*, considered as a vaginal commensals in healthy individuals [17] and *Escherichia-Shigella.*

#### Impact of route of delivery on transmission of ASVs from mother to neonate

For each mother-neonate pair, we calculated the amount of ASVs that were shared between maternal swabs and neonatal fecal samples. Mode of delivery was taken into account in order to assess the process of early maternal seeding. From the shared ASVs, the relative abundance of each ASV was determined in the maternal vaginal and rectal swab and in the neonatal swab. This was done for all three time-points.

**Fig. 3:**
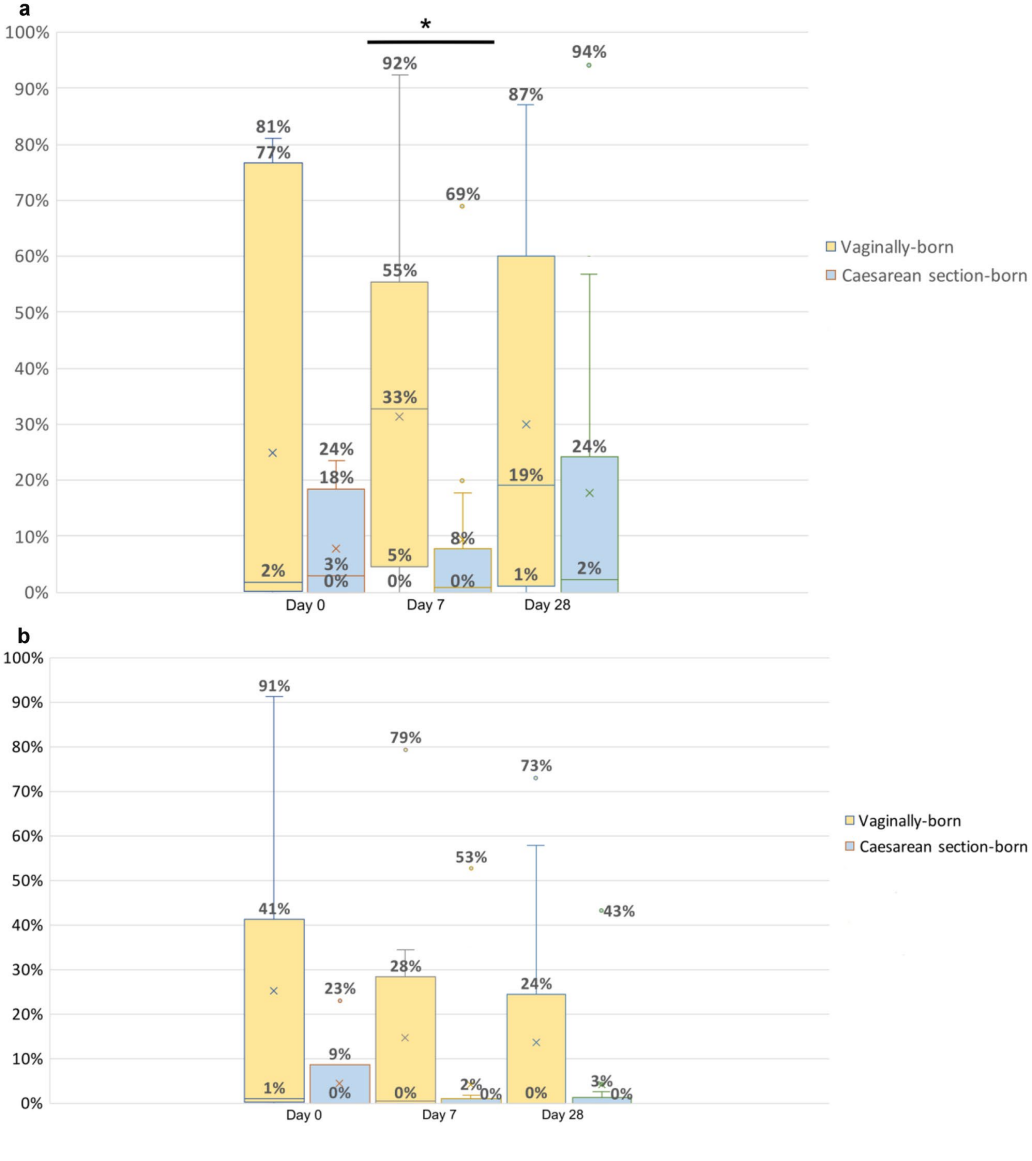
**a** Relative abundance of shared ASVs between maternal rectal swabs and neonatal samples (vaginally-born versus caesarean-section born). Day 0: p-value = 0.731, **Day 7: p-value = 0.002**, denoted by the asterisk, Day 28: p-value = 0.095. **b** Relative abundance of shared ASVs between maternal vaginal swabs and neonatal samples (vaginally-born versus caesarean section-born). No significant differences were found. Day 0: p-value = 0.295, day 7: p-value = 0.128, day 28: p-value = 0.395

On day 0 and day 28, there were no significant differences (p-value day 0 = 0.73, p-value day 28 = 0.095) in the relative abundance in neonatal samples of ASVs that were shared with maternal rectal swabs between vaginally-born neonates (represented in yellow bars in Fig. 3A) (median 2% (IQR 0–77%, range 0–81%),) and median 19% (IQR 1–60%, range 0–87%) respectively) and CS-born neonates (represented in blue bars in Fig. 3A) (median 3% (IQR 0–18%, range 0–24%), median 2% (IQR 0–24%, range 0–94%) respectively). On day 7 there was a significant difference (*p* = 0.002) in the relative abundance in neonatal samples of ASVs that were shared with maternal rectal swabs between vaginally-born neonates (median 33% (IQR 5–55%, range 0–92%) and CS-born neonates (median 0% (IQR 0–8%, range 0–69%).

On day 0, 7 and 28, there were no significant differences (p-value day 0 = 0.295, p-value day 7 = 0.128, p-value day 28 = 0.395) in the relative abundance of ASVs that were shared with maternal vaginal swabs between vaginally-born neonates (represented in yellow bars in Fig. 3B) (median 1% (IQR 0–41%, range 0–91%), median 0% (IQR 0–28%, range 0–79%) and median 0% (IQR 0–24%, range 0–73%) respectively) versus CS-born neonates (represented in blue bars) (median 0% (IQR 0–9%, range 0–23%), median 0% (IQR 0–2%, range 0–53%), median 0% (IQR 0–3%, range 0–43%) respectively).

## Discussion

In this study, we evaluated how maternal vaginal and rectal microbiota contribute to the early colonization process of the neonatal gastrointestinal tract, both in VB- and CS-born neonates. Only a minor part of maternal rectal and vaginal ASVs was transferred from mother to the neonate in both VB and CS-born neonates. We observed no persistent effect of mode of delivery of the number of ASVs transferred from mother to the neonates. However, in both delivery routes, the relative abundance of ASV shared with the maternal rectal swab was higher (median 19% in vaginally-born infants and 2% in CS-born infants) compared to the relative abundance of the ASVs shared with the maternal vaginal swab (0% for both vaginally- and CS-born neonates) on day 28.

As colonization of the neonatal gut is believed to start with vertical transmission of maternal microbes during delivery, the mode of delivery is initially expected to affect this process [7]. However, colonization continues after birth by horizontal transmission depending on a variety of environmental factors including exposure to medication, particularly antibiotics [4–6]. A previous study comparing stool samples from 56 neonates (28 twins), collected up to the fourth day of life, with stools from their 28 mothers, also reported no difference between shared ASVs between mothers and neonates in CS (*n* = 35) versus VB-neonates (*n* = 21) [17]. Data on microbial sharing between mothers and CS and VB-neonates is, however, conflicting. In a recent study, 72% of early gut colonizers of VB-neonates matched species in the maternal stool, whereas 41% of these taxa were seen in CS-neonates [18]. These differences could at least partly be explained by differences in methodology of the studies. Some studies included only elective CS, while other also included secondary CS and women with cervical dilation or ruptured membranes, in which cases the neonate could already have been subjected to vertical transmission from the maternal genital and gastrointestinal tract in utero. Another possible explanation is the difference in the colonization interval, meaning that the impact of CS on neonatal gastrointestinal colonization is not only immediate after birth but also postnatally by strain transmission from the hospital environment during postnatal admission [11]. Strains from the maternal gut have been shown to dominate the infant’s microbiota at four months, however over time, strain similarity to the mother declines as there is strain replacement from other, mostly horizontal, sources [11, 19]. 

Previous work has shown the association between CS and a delayed microbial maturation, as well as a lower prevalence of certain bacteria (*Bacteroides*,* Bifidobacterium*) in the infant gut persisting beyond 6 months of age, while some studies describe an effect even up to 2 years [9, 10, 20, 21]. Furthermore, infants born per CS have been reported to have a higher rate of opportunistic pathogens such as *Enterococcus*,* Klebsiella* and *Firmicutes* and a general imbalance of the gut microbiota [6, 22]. Recent work by Zhou et al.. demonstrated that vaginal microbiota transfer with a gauze containing vaginal fluids can restore gut microbiota in CS-neonates (*n* = 35) and that these neonates have significantly improved neurodevelopmental scores in comparison with CS-neonates exposed to a gauze with saline in the control group (*n* = 41) [20]. Interestingly, in a recent study including 75 newborns, the majority of neonates born per CS had ASVs belonging to the *Bacteroides* group in the first week of life which were not present in their second week of life. In 8 infants, the strain was matched with the maternal rectal swab. This disappearance suggests either a lack of an environment where *Bacteroides* thrive, a difference in colonization fitness of the species and/or the presence of an antagonist (for example, *Streptococcus*) [8]. In line with previous data, we also observed that the vaginal microbiota seems to only marginally be transferred to the neonatal gut in VB-neonates [22]. Possibly competition with other taxa influences colonization beyond initial exposure or the vaginal microbes do not thrive in the neonatal gut [8]. 

Species from the maternal gastrointestinal tract predominantly contribute to neonatal gut colonization in comparison with the maternal vaginal microbiota, regardless of mode of delivery. Previous studies have shown a range between 30 and 70% of shared proportions of the neonatal microbiota with maternal gastrointestinal sources [18, 19, 23, 24]. Drell et al. observed in a study including seven mothers (of whom five delivered per CS), the proportion of the operational taxonomic units shared between the mother’s gut microbiota and neonate’s stool microbiota was 32%, 34%, and 29%, respectively at 48–72 h, 6–8 weeks and 6 months after birth [24]. In another study that enrolled 25 women who delivered vaginally, the mothers’ stool microbiota accounted for 22.1% of the overall microbial abundance in the neonate’s gut [19]. Transmitted species that were observed included *Escherichia coli*, and gut-associated bifidobacterial species and *Bacteroides* [19]. We can only speculate about the origin of the remaining majority of the detected strains, possibly some were accumulated through horizontal rather than vertical transmission.

Maternal body sites that have been described to contribute to neonatal colonization, beside vagina, gut and skin, include the tongue dorsum and breast milk, through the bacterial entero-mammary pathway, in which the maternal gut bacteria translocate through breast milk to the gut of the infant [22, 25]. In a study involving 7 mother-neonate pairs in which all mothers delivered vaginally and exclusively breastfed, sharing of a *Bifidobacterium breve* was demonstrated in maternal fecal samples, breast milk and neonatal feces [25]. Neonatal feeding type (both breast milk and formula) influence colonization and is a significant confounder. In our study, 50% of CS-neonates and 65% of VB-neonates were exclusively breastfed. In the CS group, 23% received only formula and 28% received a combination of breastmilk and formula. In the VB group, 17% received only formula and 17% received a combination of breastmilk and formula. Other studies suggested sources for horizontal transmission of microbial species directly postpartum are for example from hospital personnel in case of CS-born neonates and strains transmitted from family members (such as the father and siblings but also pets), the environment (skin, bottle, clothes, mattress) and nutrition [19]. Strain sharing between family members has been described previously, peaking between 2 and 10 years of age and being similar between twins and non-twin siblings (ruling out a specific genetic component). Importantly, fathers introduced most new shared strains [11]. 

### Strengths and limitations

Strengths of our study include strict eligibility criteria and study protocol, taking the effect of confounders with known impact on microbiota, such as the use of antibiotics, into account. Limitations include that the average shared maternal microbiota may be an underestimation especially on day 0 since we also experienced challenges collecting and analyzing neonatal stool samples, as meconium can be difficult to sample because of its consistency. Another limitation of our study may be the use of previously sequenced data. The sequences were used for the amplicon sequence variant analyses and taxonomic assignment, however the sequences became too short to classify into genus level and as such we were left with family level. For future studies on colonization additional maternal samples from other body compartments should also be collected. Also, while neonatal feeding type, be it breastmilk or formula, presents a possible limitation since feeding type is a factor influencing colonization, in our study the confounder applies to both groups equally.

## Conclusions

We observed that only a minority of ASVs present in the maternal vaginal and rectal microbiota are transferred to the neonatal gut. The relative abundance of ASVs from the maternal rectal microbiota was greater compared to the relative abundance of ASVs from the vaginal microbiota in neonates one month of age, in both vaginally- and caesarean-born neonates. More studies need to be conducted to analyze colonization beyond 28 days postpartum and the effect of other bodily sources on colonization. Improved insight into these mechanisms may result in the development of novel microbiota-based strategies to prevent or restore early dysbiosis, which is associated with negative long-term health outcomes.

## Electronic supplementary material

Below is the link to the electronic supplementary material: Supplementary figure 1. The number of species found in each sample reached a plateau in the rarefaction curve, indicating that samples were sufficiently sequenced.

Supplementary Material 1

## Data Availability

Data is provided within the manuscript or supplementary information files.

## Abbreviations

ASVs: Amplicon Sequence Variants
VB: Vaginally Born
CS: Caesarean Section
RA: Relative Abundance
