# Supplementary figures and images for: The influence of maternal gut and vaginal microbiota on gastrointestinal colonization of neonates born vaginally and per caesarean section
Source: BMC Pregnancy Childbirth. 2025 Mar 8;25:254. doi: 10.1186/s12884-025-07358-w (PMC11889873; doi:10.1186/s12884-025-07358-w)

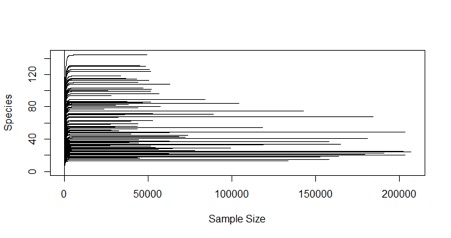

Supplement: Supplementary file 1 — Supplementary Material 1 [file 12884_2025_7358_MOESM1_ESM.jpg]
